# Monthly Summary

**Published:** 1888-02

**Authors:** 


### Monthly Summary.

Pure Mercury.—In making a dental amalgam, use always pure mercury, bought from a trustworthy dealer, who knows that the article is pure. The use of impure mercury must make a poor amalgam with the very best alloy that can be obtained. Keep the mercury constantly covered with alcohol, which should remain clear, and the surface of the mercury appear brilliantly bright. Take a common glass drop-tube with bulb; compress the bulb; pass the glass nozzle through the alcohol into the mercury; release the pressure on the bulb, and the tube will fill with mercury, which may then be dropped into the hand or mortar in as small a quantity as is desired. By this method the mercury in the tube and bottle is kept from contact with the air, and will remain bright and clean until the last. If through neglect the mercury should become oxidized, wash it by shaking it in the bottle with alcohol renewed again and again until perfectly free from discoloration by the mercury. The disesteem in which amalgam are held by many dentists and patients may be in great measure chargeable to the use of impure mercury. Every dentist experienced in the construction of silver plates knows the importance of making his solder bright and clean. Similar conditions should for like reasons be observed in making a dental amalgam with mercury, which ought not only to be pure and kept clean, but for the production of the best results it ought never to be mixed with the alloy in the hand, because of the inevitable film of dermal secretions that will be spread ovor the surface of the mercury, and by so much hinder the perfect union of the metals. The mix ought therefore to be made in a clean rubber or vitrified mortar with a rubber or glass pestle, and the pellet compressed in a piece of washed and dried buckskin. If with these precautions care is taken to keep the alloy in a tight stoppered bottle, amalgam fillings of unwonted excellence may be made.—W. S. H.

---

Chinese Anæsthetic.—A curious account of a Chinese anæsthetic is given *Nouv. Remedes* (April, p. 165). It appears that Dr. W. Lambuth mentions in his third annual report of the Soochow Hospital an experiment made, at the suggestion of a chinese doctor, with this preparation. A substance resembling wax, but harder and semi-transparent, in the form of a tablet, was cut into small pieces and digested in water for twenty-four hours, together with a small white, woody excrescence. The liquid was then found by Dr. Lambuth to possess well marked anæsthetic properties. It was found that a numbness of the lips and tongue was produced, and that the finger immersed in the solution for some minutes could then be pricked with a needle without any pain being felt. The tablet was described as being the juice of the eyes of a frog. It was probably the substance obtained by the Chinese by placing a frog in a jar containing flour and irritating the animal, when it exudes a liquid which forms a paste with tne flour. This is then dried and made into cakes bearing some resemblance to button lac. If the anæsthetic property be due to the frog's excretion, and not to the white, woody excrescence above mentioned, the facts suggests the possibility of the animal using the secretion to deaden the pain to which it might be subjected by its enemies.—*Extracts from Scientific American.*

---

THE CONTROL OF HEMORRHAGE.—Dr. A. Maguire says: "In antebellum days I was called to a large plantation in my neighborhood by the manager, one of the old-time overseers, who had great confidence in his powers of healing, and was convinced of his superiority over any new-fledged Æsculapius, and that what he did not know of medicine was not worth knowing.

"He pointed to a ghastly looking African sitting on a veranda. with his head leaning against the brick pillar, blanched as much as his color allowed, and with a small stream of blood and saliva trickling from one corner of his mouth. He had extracted, twenty four hours before, the third molar, and the blood had never stopped. He had applied strong vinegar, Parvaz's perchloride of iron, nitrate of silver, and had caused the blacksmith of the plantation to bend and file down to a point a goodly-sized wire with which he had cauterized the socket. After doing all this he confessed he was at his wit's end. After reviving the drooping African with a square dose of whiskey, I made a wad of cotton to be compressed between his jaws, leaving a piece protruding in the mouth of sufficient size to allow the involuntary play and suction of the tongue to be exerted on it, and not disturb the formation of the clot in the socket. The hemorrhage was arrested in ten minutes.—*Pacific Record.*

---

A RECEIPT FOR MENDING BROKEN MARBLE.—Take plaster of Paris, and soak it in a saturated solution of alum, then bake it in an oven, the same as gypsum is baked to make it plaster of Paris; after which grind the mixture to powder. It is then used as wanted, being mixed up with water like plaster and applied. It sets into a very hard composition, capable of taking a very high polish, and may be mixed with various colering minerals to produce a cement of any color capable of imitating marble.—*Scientific American.*

**Mastication**—*By A. W. Troost.*—However easy of digestion food may be, it always profits by its stay in the mouth. Cases are on record of esophagean stricture in which patients have been kept alive by food introduced through a gastric fistula, and it has been agreed that the food agreed better with the patient. Better results were produced when the food was first masticated and then put into a tube in connection with the stomach. The dyspepsia so common among people who either do not or can not masticate, and consequently insalivate their food tells us this. Although nature has given us so modest a set of teeth, as compared with the size of our bodies, she does not intend them to be wholly disregarded with impunity. The more we use our teeth, the better for us and the better for them.—*Brit. Jour.*

---

**Hours of Fate.**—Dr. Richardson tells us that in the period between midnight and six in the morning the animal vital processes are at their lowest ebb. It is at these times that those who are enfeebled from any cause most frequently die. Physicians often consider these hours as critical, and forewarn anxious friends in respect to them. From time immemorial those who have been accustomed to wait and attend on the sick have noted the hours most anxiously, so that they have been called by our old writers "the hours of fate." In this space of time the influence of the life-giving sun has been longest withdrawn from man, and the hearts that are even the strongest beat with subdued tone. Sleep is heaviest and death is nearest to us all in "the hours of fate."

---

**Difference Between Creosote and Carbolic Acid.**—Creosote is a distillation from wood tar, carbolic acid from tar of mineral coal; creosote is an oil, carbolic acid an alcohol; creosote is an non-crystalizable fluid, carbolic acid in its pure state, is always crystalized, except when quite warm; creosote is not soluble in water, carbolic acid is; creosote is not a caustic, carbolic acid is a powerful caustic; creosote is not a germicide, carbolic acid is.—*Mat. Med. and Phar.*

---

**Mercurial Paralysis**—While investigating the effects of poisoning by mercurial salts, Mr. Maurice Letulle found that in the lesions affecting the nervous substance, the myeline sheath disappears rapidly and the cylindral axis, on the contrary, persists. It seems even to persist indefinitely, and this is what explains why mercurial paralysis heals very rapidly.—*Ex.*

---

**If** all the operative dentists of a city could be prevailed upon to close their offices upon Saturday afternoons and spend the half day in the pursuit of health and pleasure, each and every one of them at the end of the year would find himself the better for it, not only physically but financially.—*Extract Editorial from Independent Practitioner.*

